# Influence of Different Alloying Strategies on the Mechanical Behavior of Tool Steel Produced by Laser-Powder Bed Fusion

**DOI:** 10.3390/ma14123344

**Published:** 2021-06-17

**Authors:** Abootorab Baqerzadeh Chehreh, Anna Strauch, Felix Großwendt, Arne Röttger, Rainer Fechte-Heinen, Werner Theisen, Frank Walther

**Affiliations:** 1 Department of Materials Test Engineering, TU Dortmund University, Baroper Str. 303, 44227 Dortmund, Germany; frank.walther@tu-dortmund.de; 2 Leibniz Institute for Materials Engineering, Badgasteiner Str. 3, 28359 Bremen, Germany; strauch@iwt-bremen.de (A.S.); fechte@iwt-bremen.de (R.F.-H.); 3 Chair of Materials Technology, Ruhr-University Bochum, Universitaetsstr. 150, 44780 Bochum, Germany; felix.grosswendt@ruhr-uni-bochum.de (F.G.); Arne.Roettger@ruhr-uni-bochum.de (A.R.); theisen@wtech.rub.de (W.T.); 4 Chair of New Manufacturing Technologies and Materials, Bergische Universitaet Wuppertal, Bahnhofstr. 15, 42651 Solingen, Germany; 5 MAPEX Center for Materials and Processes, University of Bremen, 28359 Bremen, Germany

**Keywords:** powder mixing, new alloying strategies for additive manufacturing, tool steel, laser-powder bed fusion (L-PBF), compression test, computed tomography

## Abstract

Additive manufacturing is a high-potential technique that allows the production of components with almost no limitation in complexity. However, one of the main factors that still limits the laser-based additive manufacturing is a lack of processable alloys such as carbon martensitic hardenable tool steels, which are rarely investigated due to their susceptibility to cold cracking. Therefore, this study aimed to expand the variety of steels for laser powder bed fusion (L-PBF) by investigating an alternative alloying strategy for hot work tool steel powder. In this study, a comprehensive investigation was performed on the powder and L-PBF processed specimen properties and their correlation with the existing defects. Cubical specimens were created using the following two alloying strategies by means of L-PBF: conventional pre-alloyed gas-atomized powder and a mixture of gas-atomized powder with mechanically crushed pure elements and ferroalloys. The influence of the particle parameters such as morphology were correlated to the defect density and resulting quasi-static mechanical properties. Micromechanical behavior and damage evolution of the processed specimens were investigated using in situ computed tomography. It was shown that the properties of the L-PBF processed specimens obtained from the powder mixture performs equal or better compared to the specimens produced from conventional powder.

## 1. Introduction

Additive manufacturing (AM) enables the possibility to create near net shape parts without the need for further assembling or post-process joining and almost with no limits regarding the geometrical complexity [1]. Materials densified by AM technologies can achieve tensile strengths equal to or even better than those obtained via conventional manufacturing processes [2]. In addition, AM technologies present an ecological and economic solution for products improvement and sustainability by enabling the repair and refurbishment of components with minimal material usage [2,3,4,5,6,7]. Therefore, this technique soon became a suitable alternative to conventional casting routes for medium to low-batch productions in automotive, aerospace, health care, and consumer goods industries [6].

Among the AM techniques, laser-powder bed fusion (L-PBF) of metallic materials has been used most extensively during the last decade by researchers as well as different industries [8]. In L-PBF, powder (feedstock) layers are selectively melted by a focused laser beam. This layer-wise build-up is repeated multiple times until the manufacturing process of the part is finished [9]. Based on the mentioned advantages, the L-PBF process allows for the production of complex-shaped tools with optimal geometries [10], which are exceedingly time and energy consuming using conventional manufacturing processes. However, during the L-PBF process, strong heat flux from the melt pools into the already solidified parts leads to high heating and cooling rates, which promote the formation of cold cracks in carbon martensitic steels due to the high thermal stresses [11]. It requires adapted tool steels to avoid this problem. However, the development of new alloys for L-PBF is not a simple task due to the limiting final part quality and, usually, it requires a high economical investment that ultimately increases the price of final parts [12,13]. That is the reason why there are only few processable materials by the L-PBF technique, which are mostly aluminum (AlSi12, AlSi10Mg) and titanium alloys (TiAl6V4) [14,15]. There are only a few Fe-based alloys among the processable materials, which have been investigated so far. They are mostly austenitic stainless steels (e.g., 316L/304L) and maraging steels (18Ni-300) with relatively new focus on carbon martensitic hot-work tool steel H13 [13,16]. High-performance materials such as hot-work tool steels are crucial for hot-work applications such as forging, die casting, cutting, etc. which makes them an interesting topic for additive manufacturing. However, due to the several process difficulties such as crack-inducement during the martensitic transformation, they have not been extensively studied [6].

On another hand, powder morphology is a critical factor in the L-PBF process, since it generally requires highly spherical gas-atomized powders with specific particle size distribution to guarantee a stable process and a suitable defect-free final product [17]. The powder properties have a direct effect on the melt pool behavior, the thermal conductivity, and the laser energy absorption [18,19]. This requirement significantly limits the accessibility of commercially available materials for L-PBF-processing, since only a few companies are able to produce suitable gas-atomized powders.

An alternative to the existing pre-alloyed powders that can greatly reduce the production cost is the fabrication of metal alloys mixed with pure elemental powders, which only few studies have paid attention to [3,20,21,22]. Following this methodology, the powder material can be directly modified before the AM process to influence or improve the characteristics of the final additively manufactured part. The technical feasibility of processing powder with such an alloying method has been proven for elemental mixtures containing spherical aluminum and aspherical silicon powder particles [22] and also for a powder mixture of spherical iron powder with mechanically crushed ferroalloy particles instead of using cost-intensive pre-alloyed gas-atomized powder by the authors of this study [1]. In this study, a more comprehensive investigation was performed on the mechanical properties of powder feedstock and L-PBF processed specimen and their correlation with defect density and residual stress. This work compares the new alloying technique with the conventional pre-alloyed starting material in terms of the mechanical properties of the additively manufactured specimens. This study has a special focus on the powder particle properties such as porosity (by computed tomography) and mechanical properties, which are relatively new topics and have not been comprehensively investigated before [23].

## 2. Materials and Methods

The considered carbon martensitic tool steel was selected for its low susceptibility for cold cracking, which is associated with its low martensite start temperature (<200 °C) [24]. The tool steel starting powder was produced by two methods: gas atomization of a pre-alloyed melt (called AdPre) and the mixing of crushed elemental and ferroalloy particles with gas-atomized pure iron (called AdEle).

To produce the atomized powder particles for AdPre, raw elements and ferroalloys were melted and superheated to 1710 °C in the melting chamber of a close-coupled atomizer (Indutherm, AU 1000 Prototype, Walzbachtal, Germany) flooded with argon to prevent oxidization. The atomization was performed using nitrogen gas on a melt mass flow of 290 kg/h. The iron powder for AdEle was created in the same way starting with melted bulk iron (99.9 mass% purity). This atomization has a melt mass flow of 430 kg/h. The gas-atomized powders were sieved in the fraction of 25–63 μm by an air jet sieve (Hosokawa Alpine AG, Air Jet Sieve e200LS, Augsburg, Germany).

The crushed particles in AdEle include Cr, Ni, Mn, FeCrC (22/70/8 mass%), FeMo (70/30 mass%), FeSi (25/75 mass%), FeTi (30/70 mass%), FeV (20/80 mass%), FeW (80/20 mass%) and were cryogenically frozen in advance to reduce the ductility while crushing. Each ferroalloy has been individually sieved (20–63 μm) and subsequently mixed by a 3D shaker mixer (WAB Group, Turbula, Muttenz, Switzerland).

The real chemical compositions of both starting materials in addition to the target composition are presented in Table 1. The carbon content was separately measured using carrier gas hot extraction (Bruker, IR07, Billerica, MA, USA) due to experimental restrictions concerning lightweight elements in energy-dispersive X-ray diffraction. Both achieved alloys showed a good approximation to the targeted chemical composition.

### 2.1. Powder Feed Stock Characterization

**Figure 1:**
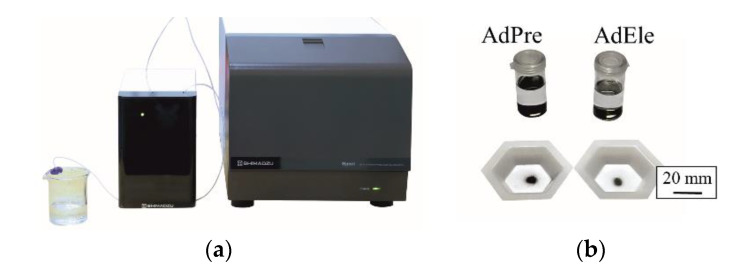
(**a**) iSpect DIA-10 system and (**b**) sample solution for the dynamic image analysis.

**Figure 2:**
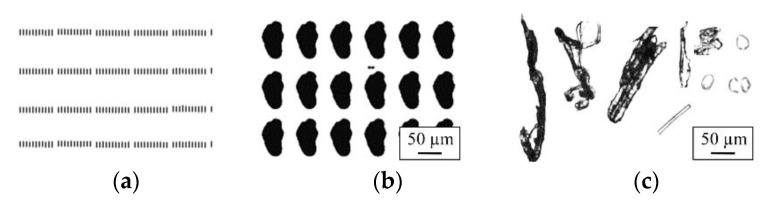
(**a**) Measurement noises, (**b**) powder particle repetitions, and (**c**) foreign particles detected during the dynamic image analysis.

The particle size distributions, powder morphology, and circularity of the powders were analyzed with an iSpect DIA-10 (Shimadzu, Kyoto, Japan) (Figure 1a). This is a dynamic image analysis system that enables particle detection with an accuracy and reproducibility of ±5% through microcell technology that gives it an efficiency of more than 90%. About 5 mg from each powder was mixed into 5 mL of ethanol to create a sample solution for the powder characterization (Figure 1b). Then, 200 µL (minimum required volume is 50 µL) of the sample solution was loaded in the iSpect DIA-10 and analyzed. This system provides the images of every single analyzed powder particle. Therefore, after the measurements, noises (Figure 2a), repetitions in measurements (Figure 2b), and foreign particles (Figure 2c) were filtered out from the results.

Particle morphology has also been investigated by microscopy, for which the powder particles have been embedded on a conductive adhesive tape. To investigate the cross-section of the powder particles, they were embedded in an electric conductive resin, mechanically ground with SiC abrasive paper with 320, 800, and 1000 mesh, and finally polished using a diamond suspension liquid with particle sizes of 6, 3, and 1 µm. Afterwards, the powder morphology and the cross-sections of the powder particles were investigated with the help of a scanning electron microscope (SEM) type Mira 3 (Tescan, Brno, Czech Republic) using the secondary electron detector, an acceleration voltage of voltage UA = 20 kV and a working distance of WD = 10 mm.

Microcomputer tomography (μ-CT) was performed on the powder particles in order to reach a more comprehensive understanding on the existing defects. Prior to the μ-CT investigations, the powders have been poured into a cylindrical glass container with an internal diameter of approximately 800 µm. The powder was tapped and compressed from the top using a flat-headed pin to fixate it and minimize the gap between the powder particles. Afterwards, a rubber cover was placed on the container to hinder the movement of the particles during the scan. The μ-CT system type XT H 160 (Nikon, Tokyo, Japan) equipped with a microfocus X-ray source (tungsten) with a maximum voltage of 160 kV and 3 µm threshold for 3D scan was used for the investigations. All the relative scanning parameters for the CT investigations of the powder particles are mentioned in Table 2.

After the powders have been scanned, the 2D images were reconstructed into a 3D image using the CT Pro 3D software (Nikon, Tokyo, Japan). Subsequently, it was loaded into the analyzing and visualizing software VGStudio Max 2.2 (Volume Graphics, Heidelberg, Germany). By using the “Enhanced (v2.2)” algorithm, pores were detected from the specific voxels based on their gray value in comparison to a defined local threshold for contrast. The accuracy of the CT scans was ensured by adjusting and manually optimizing the gray values for correct surface detection of the powder particles. Afterwards, 2D images from the scan were controlled before the defect analysis. In addition, the minimum effective pixel size and detection probability was set to 3 µm, according to the systems detection capacity.

### 2.2. L-PBF Fabrication

Cubic samples with a dimension of 4 × 4 × 6 mm^3^ were produced by means of L-PBF using an AconityMINI system (Aconity, Herzogenrath, Germany). Specimens were built on a 140 mm diameter stainless steel type 304 platform without support structures. For the LPBF process, argon gas atmosphere with a purity of 99.999% and a maximum of 10 ppm impurities was used. The LPBF machine was inerted, and the LPBF process started when an oxygen content <20 ppm was measured. However, the oxygen drops to <0 ppm within the first layers.

**Figure 3:**
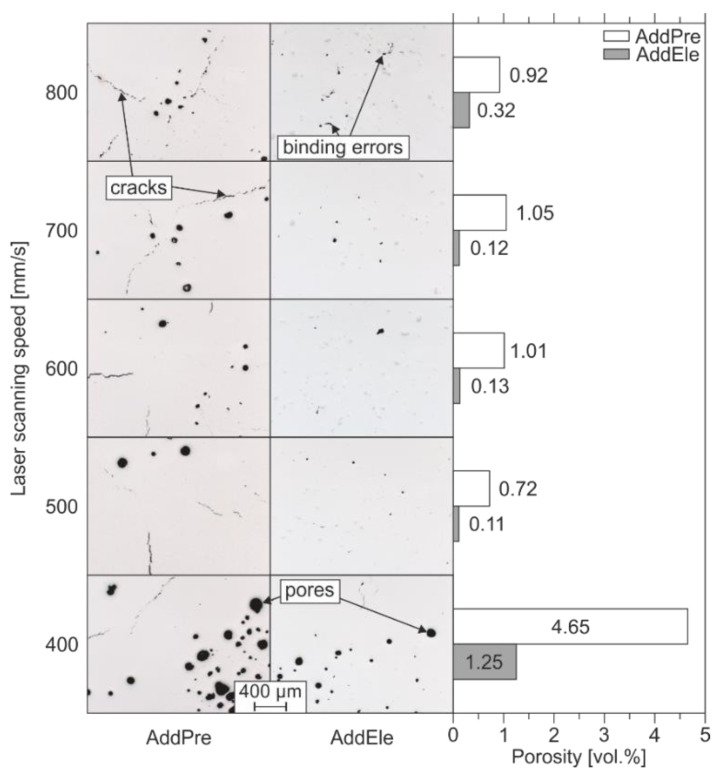
Process parameter investigation and reference porosities.

Based on the results obtained in the previous work [3], the laser power in the L-PBF process was set to 250 W. Nonetheless, to find optimized LPBF parameters for the densification of the AddPre and AddEle feedstock, a parameter study was performed by varying the laser scanning speed between 400 and 800 mm/s. For both the pre-alloyed and the mixed starting powders, samples with the lowest porosity were achieved by applying a scanning speed of 500 mm/s. Lower scanning speeds and therefore higher energy inputs result in an increased number of spherical pores. These can be associated with the evaporation or outgassing of chemical elements due to an excessive heat input. At higher scanning speeds and respectively lower energy inputs, the AddEle samples suffer from irregularly shaped binding defects due to an insufficient wetting behavior. The process parameter study and the reference porosity can be seen in Figure 3. To determine the porosity of the L-PBF densified samples of the parameter study, quantitative image analysis was applied. Therefore, optical micrographs were taken in three different planes of the polished samples cross-sections. An Olympus BX60M light optical microscope with a 50-fold magnification was used in the brightfield mode. The micrographs were binarized using the java-based software imageJ (version 1.52p, Bethesda, MD, USA) to calculate the pore volume.

All L-PBF densified samples investigated in the following were produced with the optimum parameter set for both feedstock consisting of a laser power of 250 W and a laser scanning speed of 500 mm/s. All relevant scanning parameters are shown in Table 3.

### 2.3. Residual Stress

**Figure 4:**
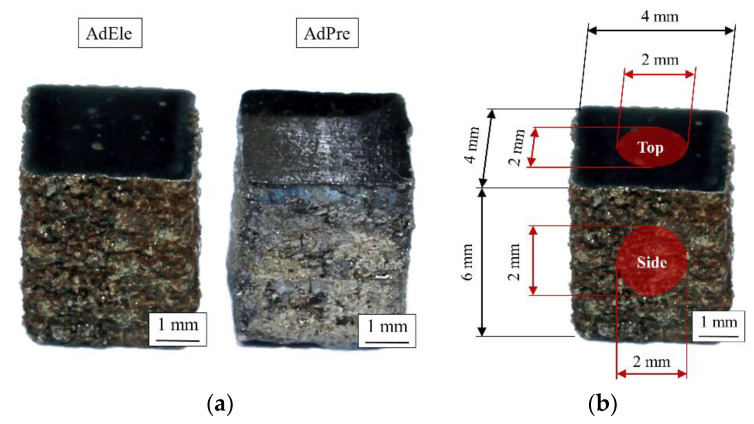
(**a**) Polished L-PBF processed specimens and (**b**) residual stress measurement areas.

The investigation of the residual stress state has been conducted using X-ray diffractometry (XRD). An X-ray diffractometer (D8 Discover, Bruker, Billerica, MA, USA) with a copper X-ray tube inside inclination and Bragg–Brentano geometry with the sin²ψ method have been used for these investigations. The residual stress states were analyzed at the center of the top surface of the specimens in as-built and polished condition and the as-built state of side surface in a circular area with diameter of 2 mm (Figure 4b).

### 2.4. Metallography and Microscopy

CT investigations were performed on the L-PBF processed specimens in order to have comprehensive insight about the process-induced porosity and to obtain valuable information about the pore size and morphology and the statistical distribution of the pore characteristics. All the relative scanning parameters for the CT investigations of the L-PBF processed specimens are mentioned in Table 4.

Then, 2D images were reconstructed into a 3D image after the scan using the same method as the scan of the powder particles. For each material, three specimens were scanned.

**Figure 5:**
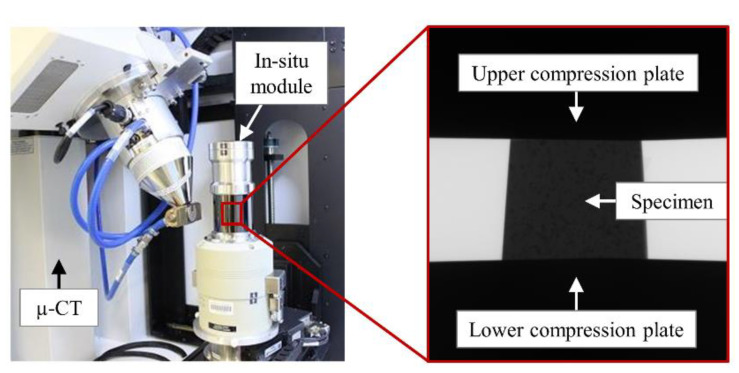
In situ CT testing setup.

To investigate the material behavior more extensively, in situ CT has been carried out in addition to the conventional CT. In situ CT enables the study and recording of material and damage behavior as a function of an applied load. In this respect, an AdEle specimen was grinded and polished from all sides equally to produce an approximately 1.8 × 1.8 × 1.8 mm³ cube that was subsequently tested under quasi-static compressive loading in the in situ CT stage (Deben Uk, CT5000, Fmax = ±5 kN) (Figure 5) at four different load levels of 0, 1, 3, and 5 kN. The force between the lower and upper clamping device was transmitted on the cubic specimen by a homogeneously constructed glass-like carbon tube with thickness of 3 mm that allows X-rays under a small uniform damping. The scanning parameters for the in situ CT investigations were the same as conventional CT investigations with effective pixel size (5.4 µm) as the only difference.

### 2.5. Mechanical Testing

Quasi-static compression tests have been carried out on the single powder particles of each material to investigate the correlation between the mechanical properties of the powders and the final L-PBF processed specimens. Tests have been performed using an ultra-micro hardness testing system (DUH-211S/MCT-W, Shimadzu, Kyoto, Japan) with a maximum load capacity of 1900 mN. The system was equipped with a flat indenter with a diameter of 50 µm and two cameras (up view and side view) that were used to find and position the powder particles exactly under the indenter. The particles were dispersed on a cemented tungsten carbide plate that was polished down to a surface roughness of <1 µm. The compression tests were performed with a speed of 70 mN/s and stopped after 1000 mN was reached. Three different powder particles were tested for AdPre and for all of the ferroalloys and elements within the AdEle powder (Fe, Ni, Cr, Mn, FeMo, FeSi, FeV, FeW, FeTi, and FeCrC).

The mechanical properties and deformation behavior of the L-PBF processed specimens were investigated as well by quasi-static compression tests at room temperature using a servohydraulic testing system (Instron PSB100, Instron 8800 Controller, Instron, Norfolk, MA, USA) with a 100 kN load cell. Compression tests were performed according to DIN 50106, and the applied load was parallel to the building direction in L-PBF. The end-face surfaces of the specimens were polished down to Rz ≤ 6 µm and were greased with lubricant to minimize the friction between the cuboid specimen and compression dies. The polishing process reduced the height of specimens from 6 mm to approximately 5.3 mm. A tactile extensometer (Instron, Norfolk, MA, USA) with a gauge length of 12.5 mm and a strain measurement range of ±40% was mounted on the slots on the compression dies for the strain measurement. The quasi-static compression tests were strain controlled, and they were stopped at a maximum applied load of 21 kN. For each material, three specimens were tested.

## 3. Results and Discussion

### 3.1. Powder Size Distribution and Morphology

**Figure 6:**
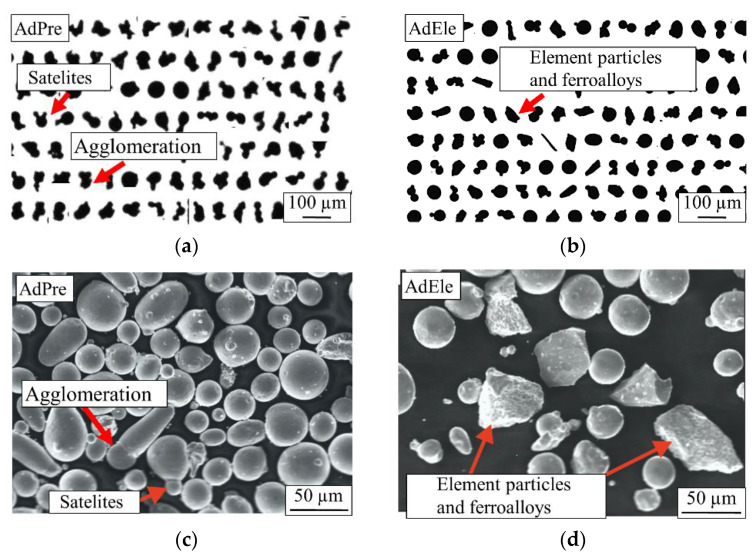
AdPre powder particles: (**a**) Dynamic image analysis and (**c**) SEM micrograph; AdEle powder particles: (**b**) Dynamic image analysis and (**d**) SEM micrograph.

**Figure 7:**
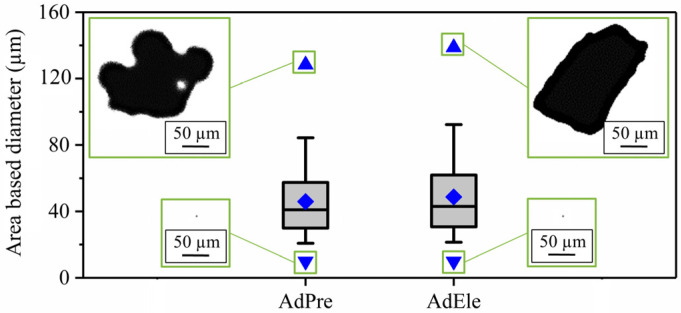
Powder size range of AdPre and AdEle powder particles.

In total, 244,520 AdPre and 263,801 AdEle powder particles were investigated using dynamic image analysis. As it can be seen in Figure 6a, a significant amount of AdPre powder particles were agglomerated or had satellites adhering, which is associated with the gas atomization process. Figure 6b shows the shapes of AdEle powder particles, which include spherical atomized iron particles and irregular-shaped crushed elemental and ferroalloy particles. SEM micrographs of both materials (Figure 6c,d) validated the mentioned observations [3]. As can be seen in Figure 7, AdEle had a bigger mean particle size, a larger maximum powder particle size, and a slightly wider particle size range than the AdPre powder.

**Figure 8:**
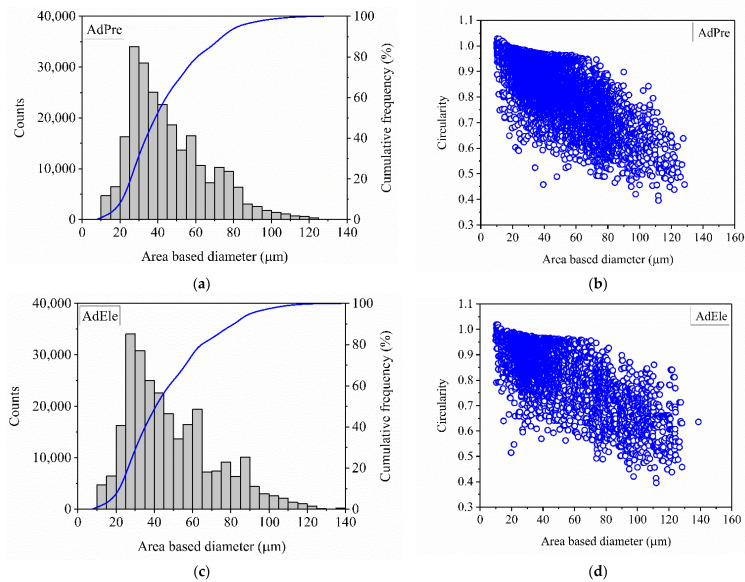
Powder size distribution of the (**a**) AdPre and (**c**) AdEle powders. Circularity of (**b**) AdPre and (**d**) AdEle powders.

Figure 8a,c show that most of the powder particles has a size between 20 and 80 µm for both AdPre and AdEle starting powders. The count numbers shown in the plots were made after filtering out the invalid measurements shown in Figure 2.

It can be seen that both powders are highly spherical up to the 60 µm diameter (Figure 8). For larger-sized particles, the circularity of both AdPre and AdEle powders drops significantly. This is due to the fact that the larger powder particles were either agglomerated (AdPre) or crushed particles with sharp edges (AdEle).

However, the decrease in the circularity of the AdPre powder seemed to be more pronounced due to a high fraction of agglomerates and satellite afflicted particles. The most important characteristic properties of both powders are depicted in Table 5. This table summarizes the powder size and morphology investigations in this work as well as the flowability analysis performed in the previous study by the same authors [3]. The powder particle size parameters of d_10_, d_50_, and d_90_ for AdPre were 24.1 μm, 41.1 μm, and 76.7 μm, and for AdEle, they were 25.8 μm, 43.9 μm, and 84.3 μm, respectively. d_min_ detected for both allowing strategies was 9.9 μm; however, d_max_ for AdPre and AdEle powder particles were 128.3 μm and 138.3 μm, respectively.

### 3.2. Defect Analysis of Powder Particles

**Figure 9:**
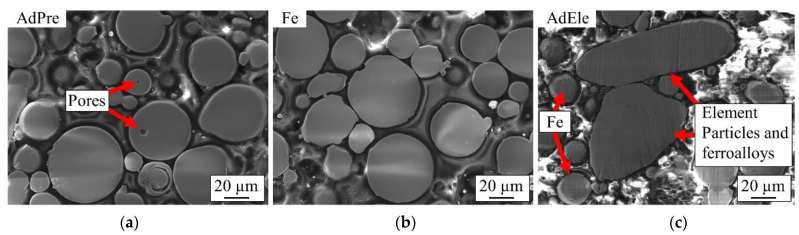
SEM micrographs of cross-sections of the (**a**) AdPre, (**b**) atomized Fe, and (**c**) AdEle powders.

The cross-sections of the feedstock powder particles were investigated using SEM. Within the AdPre powder, which was produced fully by the atomization process, there existed several small pores with diameter of less than 10 µm (Figure 9a). These small pores were most probably shaped during powder production and contain trapped atomization gas [3,25]. These trapped gasses within powder particles can remain in the L-PBF processed specimens and create unwanted defects [3,26]. On the other hand, the crushed powder particles in AdEle were completely dense (Figure 9c). Even within the atomized Fe particles, which comprise the majority of the AdEle powder, almost no porosity could be detected (Figure 9b).

**Figure 10:**
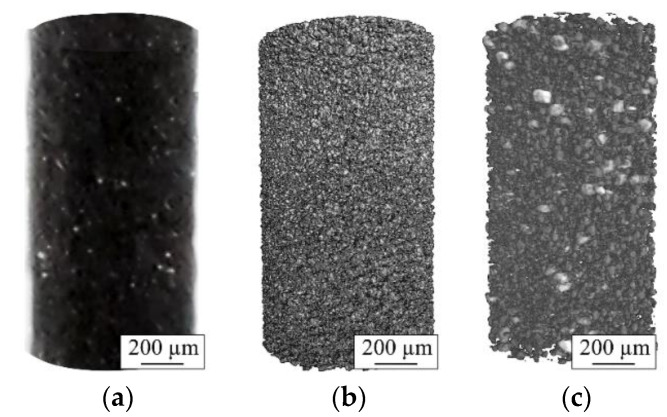
(**a**) Prepared cylindrical powder sample for the CT investigation and the 3D images of the scanned (**b**) AdPre and (**c**) AdEle powders.

Figure 10 shows the 3D reconstructed images from the µ-CT scans performed on the AdPre and AdEle powder particles. As can be seen, the AdPre powder consisted of relatively finer particles in contrast to the AdEle powder, which included multiple bulky elements and ferroalloy particles with sharp edges. The bright particles (specially in AdEle) are the tungsten-rich particles that create a lower gray scale due to their high density.

**Figure 11:**
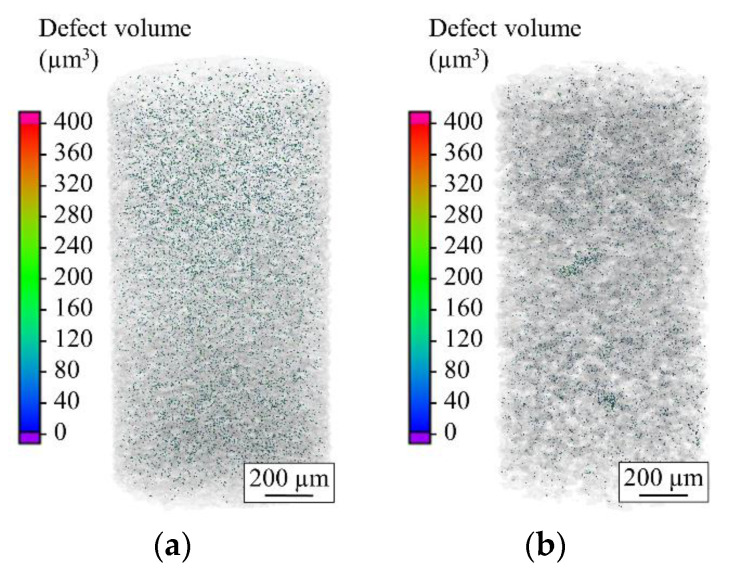
Defect analysis of the (**a**) AdPre and (**b**) AdEle powder particles.

A 8.5 × 10^8^ µm^3^ and 8.2 × 10^8^ µm^3^ volume of powders has been scanned for AdPre and AdEle, respectively. As indicated by the SEM micrographs, the µ-CT scans show a higher relative density of 99.97% for AdEle in comparison to the AdPre powder with 99.9% relative density (Figure 11). The maximum pore sizes detected for AdPre and AdEle powders were 372.9 and 290.1 µm^3^, respectively.

**Figure 12:**
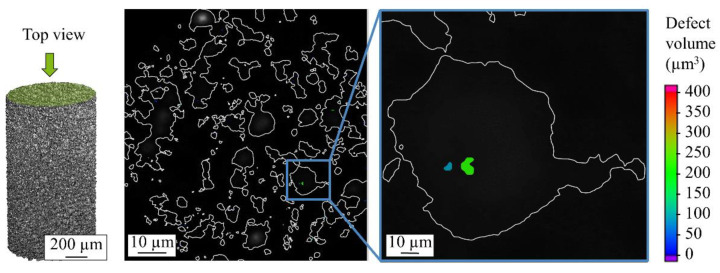
Top view 2D CT scan of the powder particles.

Figure 12 shows a top view cross-section of the scan powders. It can be seen that after the image processing, the surface detection tool properly detected and selected the perimeter of the powder particles and excluded the gaps between the particles from defect analysis considering only the voids/pores within the powder particles.

### 3.3. Mechanical Testing of Powder Particles

**Figure 13:**
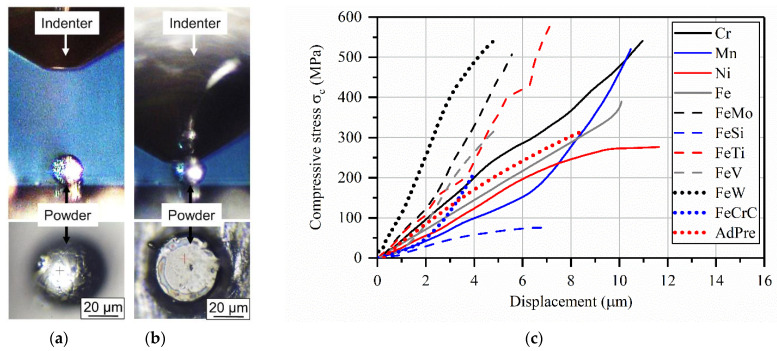
Experimental setup for the compression tests of the powder particles (**a**) before and (**b**) at the end of the test and (**c**) the final results.

Figure 13 shows the results of the compression tests on the AdPre and the elements and ferroalloy particles within the AdEle powder. For each powder, three particles have been tested, and the average of the results was plotted. Ferroalloys such as FeW, FeMo, FeV, FeTi, and FeCrC in AdEle powder showed higher slope in the stress-displacement plot than the AdPre powder particles, which clearly indicates their higher strength than the AdPre particles. On the other hand, pure elements such as Ni and Fe as well as the FeSi ferroalloy showed a lower strength than the AdPre powder. The lower strength of the AdPre powder particles in comparison to most of the particles in AdEle can partially be related to the potential existence of trapped gas within the investigated AdPre powder particles or the microstructural features, which will be investigated in future studies. For the calculation of the compressive stress, the maximum cross-section area of the powders was used. The crushed powder particles within AdEle powder contain sharp edges, which required relatively less force for deformation. After the sharp edge had been flattened by the indenter, the test force was increased at a higher rate. In order to have a reliable comparison with the AdPre powders, the relatively low initial slope in the material behavior of the AdEle powders was filtered out. It has already been shown in the previous study [3] that the L-PBF processed parts contain elemental segregations and unmolten particles. These inhomogeneities can subsequently affect the properties of the processed part such as hardness and strength. The mechanical properties of the feedstock powder particles, which contain similar elemental and ferroalloy compositions that are segregated or remained unmolten within metallic matrix after processing, can be a good representation of those inhomogeneities and later be correlated with the mechanical properties of the L-PBF processed part.

### 3.4. Defect Analysis of L-PBF Specimens

**Figure 14:**
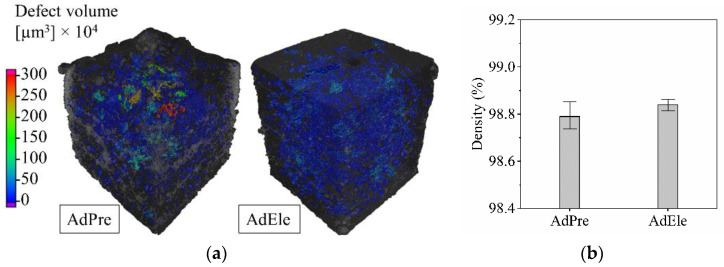
(**a**) Three-dimensional (3D) CT scans and (**b**) relative densities of AdPre and AdEle L-PBF processed specimens.

**Figure 15:**
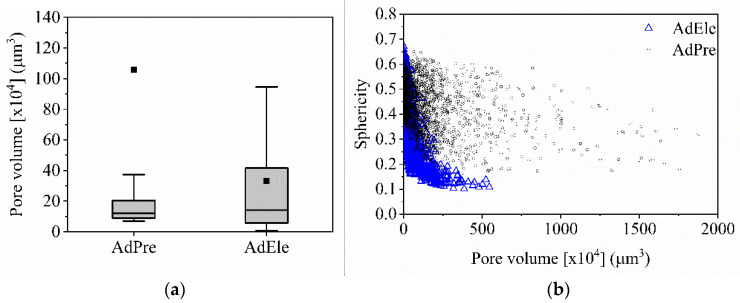
(**a**) Pore volume distribution and (**b**) sphericity of AdPre and AdEle L-PBF processed specimens.

CT investigation of the L-PBF processed specimens showed that AdPre and AdEle specimens have an average relative density of 98.79% and 98.84%, respectively (Figure 14). It can be seen that AdEle specimens contain higher number of defects; however, the existence of significantly large pores in AdPre specimens lower the relative density in comparison to the AdEle specimens. The maximum pore volumes in AdPre and AdEle specimens are 2.8 × 10^7^ and 5.4 × 10^6^, respectively. It can be seen in Figure 15a that AdEle specimens possess a wider pore size distribution range but have significantly lower average pore sizes. The sphericity of the pores decreases with the increase of the pore volume, which is more visible in case of AdEle specimens (Figure 15b).

The slight reduction in relative density of AdPre L-PBF specimens can be traced back to a higher portion of gas pores (d < 20 µm according to CT) included in the AdPre starting powder particles. It was shown by CT and SEM investigation of the powder particle’s cross-sections that less atomized powder particles contain a lower proportion of powders with trapped gas within the powder particles. However, the pore size in the L-PBF processed specimens can be much bigger than the porosities detected within the powder particles. Therefore, this is probably not the only reason for the increased porosity in the AdPre L-PBF processed specimens. A probable explanation can be that the large pores have a lack of fusions due to flowability issues caused by the satellites and sharp-edged particles.

**Figure 16:**
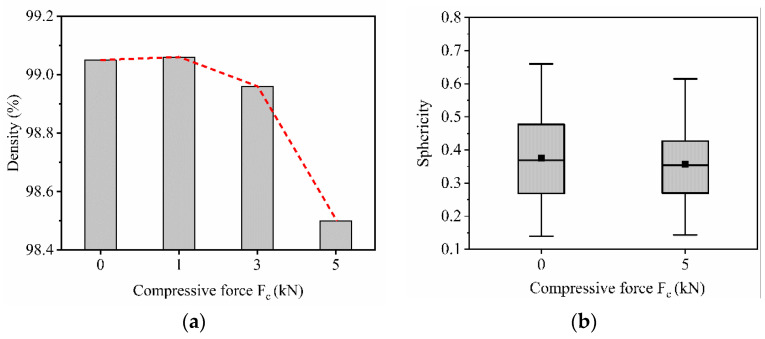
(**a**) Relative density and (**b**) pore volume distribution of an AdEle specimen under compression loading.

**Figure 17:**
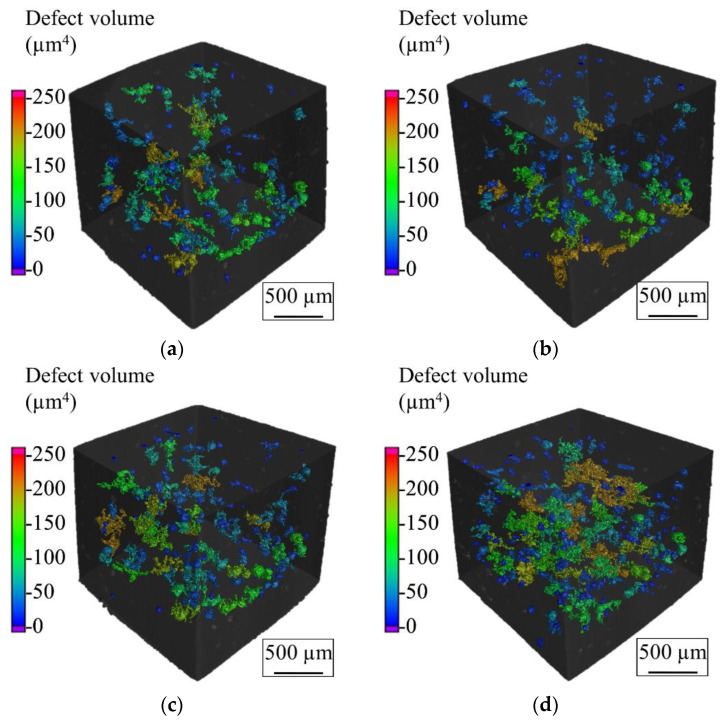
CT scans of an AdEle specimen under (**a**) 0 kN, (**b**) 1 kN, (**c**) 3 kN, and (**d**) 5kN compression loading.

**Figure 18:**
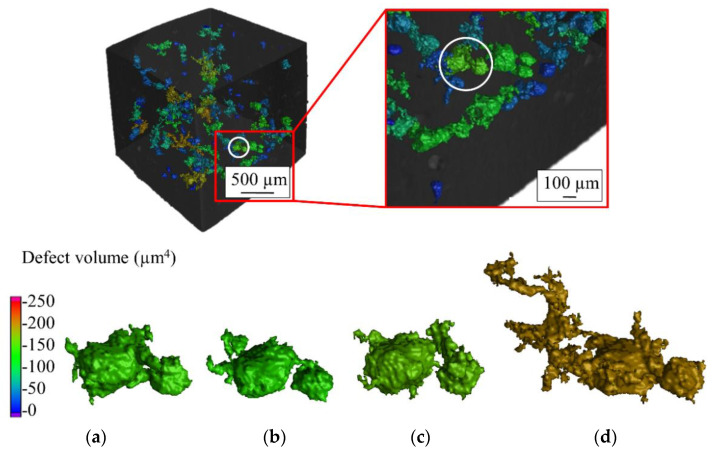
Pore size evolution of an AdEle specimen under (**a**) 0 kN, (**b**) 1kN, (**c**) 3kN, and (**d**) 5kN.

In situ CT investigation results indicated that at 1 kN compressive force (approximately 309 MPa), the density increased slightly from 99.05% (initial state at 0 kN) to 99.06% (Figure 16a). This can be explained by a slight reduction of pore volumes under compression. However, a further increase of the load to 3 kN (approximately 926 MPa) and 5 kN (approximately 1543 MPa) reduced the relative density to 98.96% and 98.5%, respectively. Due to the existence of the residual stresses within the L-PBF produced specimen and relatively low ductility of the carbon martensitic tool steel material [27,28,29], further deformation of the specimen led to the formation of additional volumetric defects such as cracks and enlargement of the existing pore volume by the expansion and formation of interconnections. The increasing number of defects and the enlargement of defects is clearly visible in Figure 17. The reduction of the sphericity of the defects under compressive load further validates the introduction of cracks as well as the flattening and connection of the existing pores (Figure 16b). As an example of the pore behavior during the compression loading, a single pore has been monitored using the in situ CT (Figure 18). An initial pore volume at 0 kN force was 1.5 × 10^6^ µm. This volume decreased to 1.4 × 10^6^ µm at 1 kN. A further increase of the compression load to 3 kN increased the pore volume to 1.6 × 10^6^ µm. The maximum volume of 2.0 × 10^6^ µm, which resembles an extreme increase of pore size by connecting to adjacent pores due to crack inducement, was reached at 5 kN. A slight flattening of the pore is also visible at 5 kN compressive load (Figure 18d). This behavior results in the reduced sphericity of the monitored pore during compressive load.

### 3.5. Residual Stress of L-PBF Specimens

The residual stress investigation of the top surface of the specimens in an as-built state by X-ray diffraction analysis showed a tensile residual stress state (Table 6). After polishing the surface as part of preparation for the compression tests, tensile residual stress was nullified, and compressive residual stress was observed on the surface of the specimens. In contrary to the top surface in the as-built state, the side surface of the specimens showed the existence of compressive residual stresses. The existing residual stress state in the as-built condition of the specimens is lower than the residual stresses reported by other studies regarding L-PBF processed carbon martensitic hot work tool steels [30]. The investigated steel is an LTT alloy (Low Transformation Temperature), which are known for compressive stresses in the as-built state as result of a martensitic transformation (volume increase of the crystals) at relatively low temperatures [31,32,33]. The volume increase during transformation is, on the basis of the present results, assumed to compensate for the residual tensile stresses introduced by the rapid cooling in the L-PBF process.

### 3.6. Mechanical Testing of L-PBF Specimens

**Figure 19:**
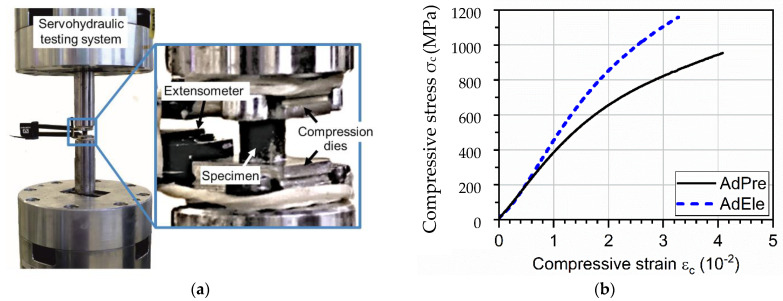
(**a**) Experimental setup for the compression tests of the L-PBF samples and (**b**) the final results.

Figure 19a shows the experimental setup for the compression tests and the mechanical properties of the L-PBF specimens. For each material, the averaged stress–strain curve of the three tested specimens was plotted. As shown in Figure 19b, the AdEle L-PBF specimens showed a higher slope in the elastic region of the stress–strain curve that translates to a higher stiffness compared to the AdPre L-PBF specimens. This is due to the fact that AdPre specimens had higher pore volume in comparison to the AdEle L-PBF specimens, since stiffness has a reverse relationship with the volumetric defect volume within a specimen. In addition, AdEle L-PBF specimens also showed a higher yield strength and hardening effect (the slope of the stress–strain curve in the plastic region) than the AdEle L-PBF specimens, which is estimated to be ground in differences in their microstructures. It has already been shown that AddPre and AddEle L-PBF specimens can possess different austenite stabilities due to elemental segregations and inhomogeneities [3]. Retained austenite can undergo a stress-induced transformation into high-strength martensite during the mechanical loading. The microstructural properties of L-PBF processed specimens using AdPre and AdEle powders will be investigated in detail in the future studies by the same authors. AdEle and AdPre had compressive strengths at a plastic deformation of 0.2% (corresponds to the compressive yield stress) of 825 MPa and 620 MPa, respectively. AdEle L-PBF samples show an approximately 33% increase in strength in comparison to the AdPre specimens. In the previous study regarding the same materials, it was found that the micro hardness of the AdEle L-PBF specimen is 37% higher than that of the AdPre specimens. However, mechanical properties (especially in the elastic region) of the specimens are also highly dependent on their relative density and average pore volume, which might be able to explain the difference in the increase rate. It is possible to identify the initiating microstructural feature or defect, which is quantified by means of size (major/minor dimension), area, perimeter, and shape factor or sphericity, respectively. Later on, the correlation between defect or the maximum stress intensity factor Kmax at the defect front and fatigue life can be described by the Murakami concept [34,35]. This will be the topic of future studies.

## 4. Conclusions

This study investigated the defect density and the mechanical properties of L-PBF processed carbon martensitic hot-work tool steel specimens produced from two different feedstock powders created by two different alloying strategies: pre-alloyed gas-atomized powder (AdPre) and a mixture of gas-atomized powder with mechanically crushed pure elements and ferroalloys (AdEle). In addition to the feasibility of the AdEle strategy, it was successfully shown that the relative density, residual stresses, and mechanical properties of the L-PBF processed specimens obtained from the powder mixture perform equal or better than the specimens produced from conventional gas-atomized powder (AdPre).

It was shown that AdEle L-PBF processed specimens contained a higher number of defects and wider pore size distribution; however, the existence of significantly larger pores in AdPre specimens lowered their relative density (98.79%) in comparison to the AdEle specimens (98.84%). This slight improvement in relative density can partially be traced back to a higher portion of gas pores (d < 20 µm) in the AdPre powder particles. Large defects in AdEle L-PBF processed specimens were mostly cracks, and on the other hand, for the AdPre specimens, it was mostly large gas pores.

Results of the compression tests on the AdPre and the pure element and ferroalloy particles within the AdEle powder showed that ferroalloys such as FeW, FeMo, FeV, FeTi, and FeCrC in AdEle powder mixtures lead to higher strength, whereas pure elements such as Mn, Ni, and Fe as well as FeSi ferroalloy caused lower strength than the AdPre powders.

The in situ compression tests in CT indicated that at 309 MPa, there was a slight increase in the relative density of the L-PBF processed specimen in comparison to the unloaded state due to a slight reduction of pore volumes under compression. However, further increase of the applied stress to 926 MP and 1543 MPa reduced the relative density. The last two loading steps led to the formation of additional defects such as cracks and enlargement of the existing pores by expansion and their interconnections and subsequent decrease of the relative density.

A tensile residual stress state has been detected on the top surface of the specimens in the as-built condition, which was transformed to compressive residual stress after polishing. On the other hand, the side surface of the specimens contained compressive residual stresses in the as-built condition, which can be associated to the volume increase during the martensitic transformation.

It was seen that AdEle L-PBF processed specimens possess a higher stiffness than the AdPre specimens due to the lower pore volume in comparison to the AdPre specimens. In addition, the yield strength of AdEle specimens was 825 MPa, which is 33% more than the AdPre ones with yield strength of 620 MPa.

In general, it can be concluded that the higher hardness [3] and quasistatic mechanical properties of the AdEle in comparison to the AdPre L-PBF specimens are caused by the high strength of the trapped unmolten ferroalloys within the AdEle L-PBF specimen’s metallic matrix and their higher relative density.

## Figures and Tables

**Table 1:** Chemical compositions in mass%.

Material	Fe	C	Cr	Si	Mn	Ni	Mo	Ti	V	W
AdPre	Balance	0.41	11.04	0.68	0.69	1.71	3.22	0.21	0.20	1.92
AdEle	Balance	0.47	9.89	0.73	0.67	1.95	3.36	0.13	0.29	2.17
Target	Balance	0.36	10.00	0.70	0.60	1.70	3.00	0.20	0.30	2.00

**Table 2:** Computer tomography parameters in scanning of the powder particles.

Beam Energy (kV)	Beam Current (uA)	Power (W)	Exposure Time (ms)	Effective Pixel Size (µm)
110	103	11.3	345	2.49

**Table 3:** L-PBF process parameters.

Laser Power (W)	Scanning Speed (mm/s)	Spot Size (mm)	Hatch Distance (mm)	Layer Thickness (mm)	Strategy	Tilt Angle (°)	Gas
250	500	0.05	0.08	0.05	Checkerboard	12	Argon

**Table 4:** Computer tomography parameters in scanning of the L-PBF processed specimens.

Beam Energy (kV)	Beam Current (uA)	Power (W)	Exposure Time (ms)	Effective Pixel Size (µm)
135	110	14.9	345	6.99

**Table 5:** Powder characteristics.

Powder Characteristics	AdPre	AdEle
Characteristic particle sizes (μm)		
d_10_	24.5	25.8
d_50_	41.1	43.9
d_90_	76.7	84.3
d_min_	9.9	9.9
d_max_	128.3	138.7
Characteristic particle shapes		
d_10_	0.69	0.68
d_50_	0.87	0.86
d_90_	0.96	0.95
Flowability [3]		
Hall flow (s/50 g)	3.2	3.6
Angle of repose (°)	62	44
Hausner ratio	1.15	1.16

**Table 6:** Residual stress measurement of as-built L-PBF processed components.

Surface	AdPre (MPa)	AdEle (MPa)
Top surface (As-built)	450	363
Top surface (Polished)	−558	−511
Side surface (As-built)	−54	−186

## Data Availability

Data sharing is not applicable to this article.
